# Genomic Surveillance Enables the Identification of Co-infections With Multiple SARS-CoV-2 Lineages in Equatorial Guinea

**DOI:** 10.3389/fpubh.2021.818401

**Published:** 2022-01-04

**Authors:** Salome Hosch, Maxmillian Mpina, Elizabeth Nyakurungu, Nelson Silochi Borico, Teodora Mikumu Alogo Obama, Maria Carmen Ovona, Philipp Wagner, Sarah E. Rubin, Ulrich Vickos, Diosdado Vicente Nsue Milang, Mitoha Ondo'o Ayekaba, Wonder P. Phiri, Claudia A. Daubenberger, Tobias Schindler

**Affiliations:** ^1^Department of Medical Parasitology and Infection Biology, Swiss Tropical and Public Health Institute, Basel, Switzerland; ^2^University of Basel, Basel, Switzerland; ^3^Laboratorio de Investigaciones de Baney, Baney, Equatorial Guinea; ^4^Infectious and Tropical Diseases Unit, Department of Medicine, Amitié Hospital, Bangui, Central African Republic; ^5^Academic Department of Pediatrics, Clinical Immunology and Vaccinology, Children's Hospital Bambino Gesù, Scientific Institute for Research, Hospitalization and Healthcare (IRCCS), Rome, Italy; ^6^Ministry of Health and Social Welfare, Malabo, Equatorial Guinea; ^7^Medical Care Development International, Malabo, Equatorial Guinea

**Keywords:** SARS-CoV-2, co-infection, variant of concern, genomic surveillance, Central-Africa

## Abstract

COVID-19 disease caused by SARS-CoV-2 represents an ongoing global public health emergency. Rapid identification of emergence, evolution, and spread of SARS-CoV-2 variants of concern (VOC) would enable timely and tailored responses by public health decision-making bodies. Yet, global disparities in current SARS-CoV-2 genomic surveillance activities reveal serious geographical gaps. Here, we discuss the experiences and lessons learned from the SARS-CoV-2 monitoring and surveillance program at the Public Health Laboratory on Bioko Island, Equatorial Guinea that was implemented as part of the national COVID-19 response and monitoring activities. We report how three distinct SARS-CoV-2 variants have dominated the epidemiological situation in Equatorial Guinea since March 2020. In addition, a case of co-infection of two SARS-CoV-2 VOC, Beta and Delta, in a clinically asymptomatic and fully COVID-19 vaccinated man living in Equatorial Guinea is presented. To our knowledge, this is the first report of a person co-infected with Beta and Delta VOC globally. Rapid identification of co-infections is relevant since these might provide an opportunity for genetic recombination resulting in emergence of novel SARS-CoV-2 lineages with enhanced transmission or immune evasion potential.

## Introduction

Whole genome sequencing of SARS-CoV-2 viruses has been widely used since the beginning of the COVID-19 pandemic to facilitate understanding of virus biology and epidemiology. The World Health Organization (WHO) recommends that countries ship at least 5% of their COVID-19 samples to reference sequencing laboratories or keep producing sequencing data if they have the capacity (1). Angola, Burundi, Cameroon, the Central African Republic, Chad, the Democratic Republic of the Congo, the Republic of the Congo, Equatorial Guinea, Gabon, Rwanda, and São Tomé and Príncipe are the 11 Central-African nations forming the Economic Community of Central African States (ECCAS). Combined, the ECCAS nations have deposited 3,924 SARS-CoV-2 whole genome sequences to GISAID and therefore have sequenced on average 0.9% of all reported cases. The proportion of sequenced cases from Central Africa over the time course of the COVID-19 pandemic reveals that with the exception of March and April 2020, the recommended sequencing rate of 5% could not be achieved.

A cost-efficient alternative to whole genome sequencing are multiplex reverse transcription quantitative polymerase chain reactions (RT-qPCR) assays which detect relevant mutations associated with SARS-CoV-2 variants of concern (VOC) (2–4). Mutation-specific assays can complement genomic surveillance programs, especially in settings where widespread sequencing capabilities are not available. If carefully designed and evaluated these kind of assays show a perfect concordance with whole genome sequencing as reported elsewhere for monitoring distinct VOC (5, 6). Mutation-specific RT-qPCR assays allow identification of patients with co-infections by more than one SARS-CoV-2 lineage simultaneously. The frequency of co-infected humans and their role in promoting SARS-CoV-2 evolution is poorly understood (7). Co-infections might provide an opportunity for genetic recombination between circulating strains resulting in the emergence of novel SARS-CoV-2 lineages (8). Interlineage recombination has been described for SARS-CoV-2 (9) as well as for other closely related viruses of the *Coronaviridae* family (10, 11).

In this brief research report, we describe the experiences and lessons learned from the SARS-CoV-2 genomic surveillance program at the Public Health Laboratory on Bioko Island, which was implemented as part of the national COVID-19 response and monitoring activities in Equatorial Guinea. We describe our approach to identify efficiently and timely SARS-CoV-2 VOC and co-infections in Sub-Saharan Africa, which is highly neglected when it comes to global genomic surveillance activities.

## Methods

### COVID-19 Datasets for Central-Africa

The number of confirmed COVID-19 cases and SARS-CoV-2 whole genome sequences from Central-Africa were obtained through the data repositories of “Our World in Data” (OWD) (12) and the “Global Initiative on Sharing All Influenza Data” (GISAID) (13), respectively on November 11^th^ 2021.

### SARS-CoV-2 Genomic Surveillance in Equatorial Guinea

Nasopharyngeal and/or oropharyngeal swab samples were collected between February 2020 and October 2021 under the umbrella of the current Equatorial Guinea SARS-CoV-2 surveillance activities. Samples that are processed at the Public Health Laboratory on Bioko Island include asymptomatic and symptomatic cases, as well as samples derived from contact tracing. Extracted RNA aliquots from positive samples are collected and stored in a local biobank at −80°C. A randomly selected subset of SARS-CoV-2 samples with Cq <30 are analyzed by spike gene mutation-specific RT-qPCR assays (*n* = 281) and/or by whole genome sequencing (*n* = 206). The spike gene mutation-specific RT-qPCR assays target three spike gene single nucleotide polymorphisms (SNPs) (L452R, E484K, and N501Y) and one spike gene deletion (Δ69/70) associated with VOCs (5, 14). In each of the four multiplex RT-qPCR assays, probes detecting the wildtype as well as probes detecting the mutant nucleotide sequences are used to enable detection of wildtype and mutated sequence simultaneously. Co-infections, with more than one single SARS-CoV-2 lineage, were defined as samples with more than one genotype in at least two of the spike gene markers analyzed. SARS-CoV-2 whole genome sequencing was conducted using the R9.4.1 flow cell on a MinION Mk1C device (Oxford Nanopore Technologies) based on the ARTIC protocol (15).

## Results

**Figure 1:**
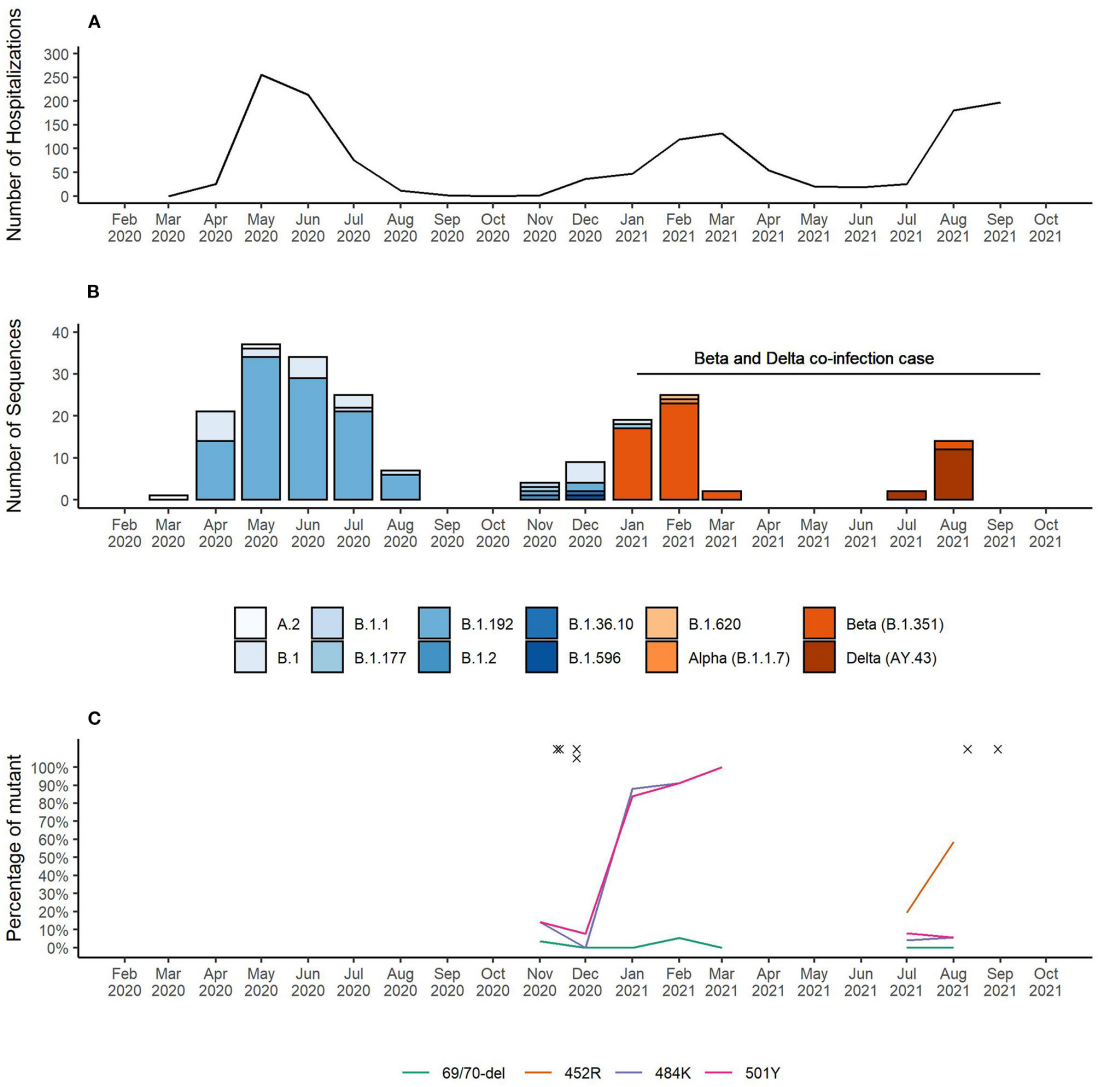
**(A)** Number of COVID-19 patient hospitalizations per month. **(B)** SARS-CoV-2 lineages identified in Equatorial Guinea over time. **(C)** Surveillance of SARS-CoV-2 VOC-associated spike gene mutations. RT-qPCR results for the spike gene mutations L452R, E484K, and N501Y and the spike gene deletion HV69/70Δ indicative of a co-infection are marked with “x”.

As of November 25^th^ 2021, Equatorial Guinea has reported a total of 13,579 confirmed SARS-CoV-2 infections, which resulted in 173 deaths. The majority (77.2%) of SARS-CoV-2 infections were reported from the insular region (Bioko Island) and 23.8% from the continental region (Río Muni) (https://guineasalud.org/estadisticas/). Since the first case was identified on March 16^th^ 2020, the country has experienced three distinct epidemic waves characterized by an increase of COVID-19-related hospitalizations (Figure 1A). Continuous sequencing of 206 SARS-CoV-2 positive samples revealed that each of these epidemic waves were dominated by a distinct lineage (Figure 1B). We showed that the first wave lasting from April to July 2020 was dominated by wildtype-like SARS-CoV-2 lineage B.1.192. The introduction of the Beta VOC (B.1.351) caused a second wave lasting from January to April 2021. The first Delta VOC (AY.43) positive cases in Equatorial Guinea were identified in the first half of July 2021 leading to a severe third wave that is still ongoing at the time of this report. Interestingly, the Alpha VOC was found only in a single sample with no indication of its widespread circulation in Equatorial Guinea. Starting from November 2020, mutation specific RT-qPCR assays were implemented to complement whole genome sequencing for enhanced genomic surveillance (Figure 1C). Three spike gene SNPs (L452R, E484K, and N501Y) and one spike gene deletion (HV69/70Δ) associated with VOCs were monitored. In each of the four multiplex RT-qPCR assays, a probe detecting the wildtype as well as the mutant nucleotide sequences are used. The 484K+501Y combination of spike gene mutation, which is associated with the Beta VOC, were first observed in November 2020 and became dominant by January 2021. This combination was later replaced by viruses with the 452R spike gene mutation, a marker for the Delta VOC. Overall, 2.1% (6/281) of samples analyzed with the spike gene mutation-specific RT-qPCR assays showed a pattern indicating co-infections of two distinct variants (Table 1). The four cases identified in November 2020, included three that had a combination indicative of co-infection between a wildtype-like lineage and the Beta VOC (wildtype and mutant specific amplification signals at spike gene positions E484K and N501Y). The fourth case showed a pattern that corresponds to a co-infection between a wildtype-like and a B.1.620-like lineage (wildtype and mutant specific amplification at spike gene positions 69/70 and E484K). Two additional co-infections were observed in August 2021, both of them with spike gene mutation patterns indicative of Beta and Delta VOC co-infections (wildtype and mutant specific amplification at spike gene positions L452R, E484K, and N501Y). All six co-infections were identified during the transition phase between two consecutive epidemic waves in which new variants emerged while other variants were still circulating.

**Table 1:** SARS-CoV-2 co-infections identified in Equatorial Guinea.

**Patient**	**Date of swab collection**	**Spike gene mutation specific RT-qPCR assay results**				Potential lineages involved in co-infection*
		**HV69/70Δ**	**L452R**	**E484K**	**N501Y**	
EG-SARS-COV-2-P1	12/11/2020	HV69/70	NA	E484 + 484K	N501 + 501Y	Wildtype (e.g., B.1.192) + Beta VOC (B.1.351)
EG-SARS-COV-2-P2	14/11/2020	HV69/70	NA	E484 + 484K	N501 + 501Y	Wildtype (e.g., B.1.192) + Beta VOC (B.1.351)
EG-SARS-COV-2-P3	25/11/2020	HV69 + 69/70Δ	NA	E484 + 484K	N501	Wildtype (e.g., B.1.192) + B.1.620
EG-SARS-COV-2-P4	25/11/2020	HV69/70	NA	E484 + 484K	N501 + 501Y	Wildtype (e.g., B.1.192) + Beta VOC (B.1.351)
EG-SARS-COV-2-P5	10/08/2021	HV69/70	L452 + 452R	E484 + 484K	N501 + 501Y	Beta VOC (B.1.351) + Delta VOC (AY.43)
EG-SARS-COV-2-P6	30/08/2021	HV69/70	L452 + 452R	E484 + 484K	N501 + 501Y	Beta VOC (B.1.351) + Delta VOC (AY.43)

*Potential lineages involved in co-infections were proposed based on spike gene mutations and known circulating variants at the time.

The receptor-binding domain of the spike protein constitutes the immunodominant target of 90% of the neutralizing activity present in SARS-CoV-2 immune sera (16). Importantly, all co-infections included one SARS-CoV-2 variant carrying the receptor-binding domain mutation E484K that has been associated with immune evasion in polyclonal human antibodies (17, 18).

**Figure 2:**
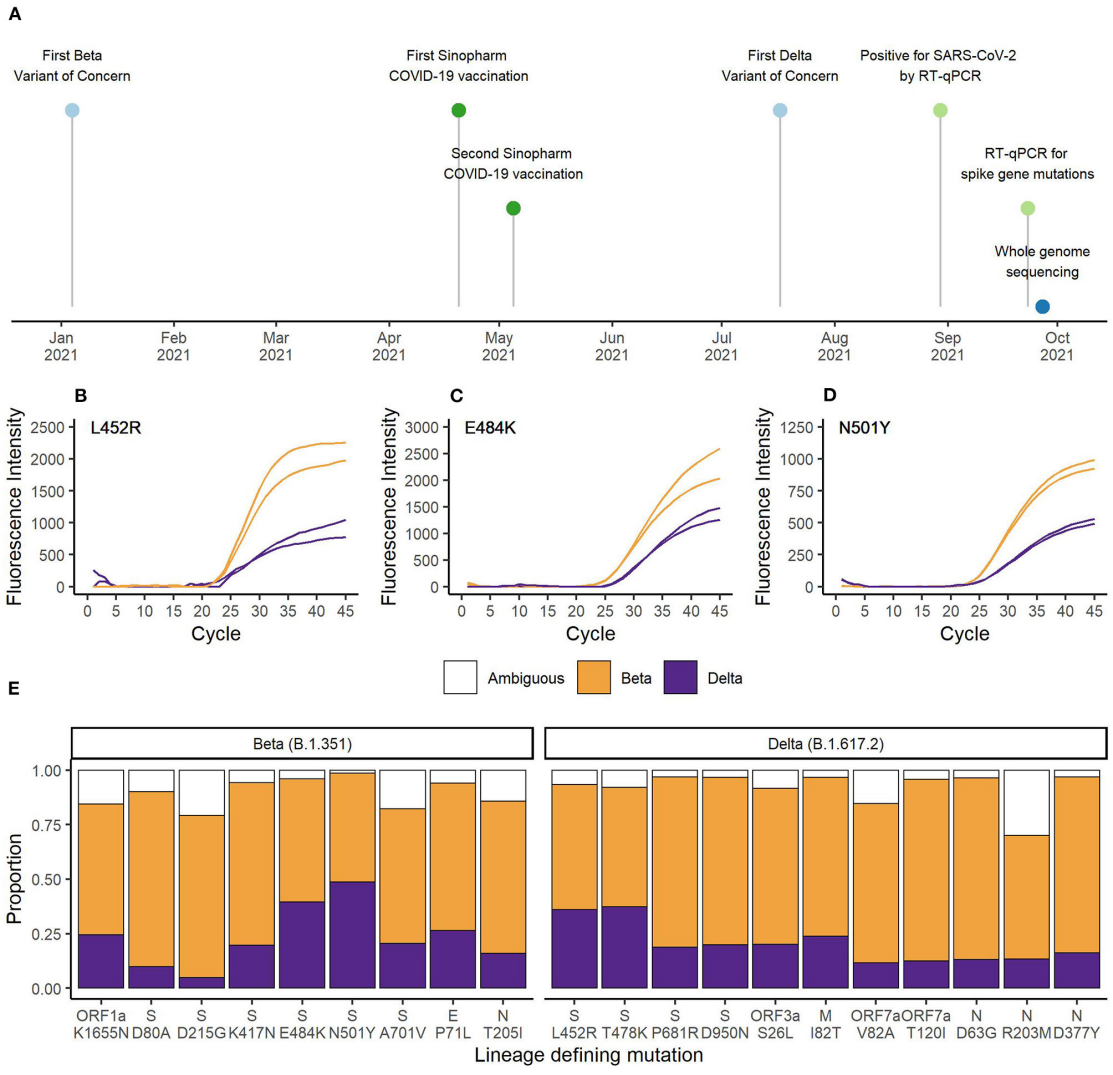
**(A)** Timeline of vaccination, diagnostic testing and whole genome sequencing. RT-qPCR results for the spike gene mutations **(B)** L452R, **(C)** E484K, and **(D)** N501Y. Orange represents the curves matching Beta VOC (L452, 484K, and 501Y) and purple represents the curves matching Delta VOC (452R, E484, and N501) defining SNPs. **(E)** Proportion of sequencing reads matching Beta or Delta VOC for the lineage defining mutations. Orange bars represent Beta VOC and purple bars represent Delta VOC. White bars represent the proportion of reads matching neither Beta nor Delta VOC.

For the EG-SARS-CoV-2-P6 co-infection case, which had the highest viral load among all co-infected patients (spike gene RT-qPCR assay Cq-value of 20.2), we were able to generate whole genome sequencing data for further investigation. The timeline of this co-infection case is shown in Figure 2A. While living under quarantine and after having been in contact with a positive SARS-CoV-2 case, a 59-year-old Equatoguinean man was tested positive by RT-qPCR for SARS-CoV-2 on August 30^th^ 2021. According to his vaccination certificate, he had received the first dose of Sinopharm COVID-19 vaccine (Beijing Bio-Institute of Biological Products Co., Ltd.) on April 20^th^ 2021 and the second dose on May 5^th^ 2021. This person had no history of clinically significant underlying medical conditions, did not report any clinical symptom during the entire period of virus infection and has reported no travel history. As part of our routine contact tracing and SARS-CoV-2 surveillance activities his sample was analyzed for the presence of three spike gene mutations associated with VOCs. The mutation-specific RT-qPCR results for the spike gene mutations are shown for L452R (Figure 2B), E484K (Figure 2C), and N501Y (Figure 2D). For all three SNPs, the wildtype as well as the mutated sequence were amplified simultaneously. An earlier and stronger amplification was observed for the mutations associated with the Beta VOC compared to the Delta VOC. In the HV69/70Δ assay, targeting a spike gene deletion common in the Alpha VOC, only the wildtype target was amplified, as neither Beta nor Delta VOC carry the deletion at positions 69 and 70 of the spike gene (data not shown).

Next, we conducted whole genome sequencing of this particular sample using our MinION sequencing platform. A total of 27,932 reads passed quality control and we achieved an average sequencing depth of 203x. Sequencing quality and depth was sufficient to identify all nine lineage-defining mutations of Beta and 11 of 12 lineage-defining mutations of Delta VOC (19). There was insufficient coverage of the spike gene SNP T19R. The average percentage of reads matching Beta VOC was 69.2% (range 50.0–83.3%) and the average percentage of reads matching Delta VOC was 21.6% (range 4.9–48.6%). Ambiguities represented 9.2% (range 1.4–30.0%) of reads. As shown in Figure 2E, the number of reads containing the mutations associated with Delta VOC was lower compared to the reads with sequences associated with Beta VOC. The higher Beta to Delta VOC ratio of RNA sample input was also observed in the RT-qPCR data (Figures 2B–D).

## Discussion

Large scale genome sequencing has become a critical part of SARS-CoV-2 surveillance globally. Yet, comprehensive analysis of genome sequences deposited to GISAID highlight stark global disparities with only 6% of genome sequences derived from low and middle income countries (20). In settings with limited access to sequencing capabilities, targeted sampling with low coverage of positive cases might be a possible alternative to representative sampling which would enable population-based surveillance. The Equato-Guinean Public Health Laboratory located in the Baney district of Bioko Island uses a hybrid approach combining rapidly adaptable spike gene mutation-specific RT-qPCR assays and whole genome sequencing to monitor the emergence, evolution, and spread of SARS-CoV-2 lineages in the country. Selection of samples for whole genome sequencing can be guided by the results of the mutation-specific RT-qPCR assays and has successfully led in the past to the discovery of the novel lineage B.1.620 (21). Additionally, results from mutation-specific RT-qPCR assays can be generated in real-time in standard local diagnostic laboratories and therefore genomic surveillance becomes actionable. Before implementing mutation-specific RT-qPCR assays, it took more than 8 weeks between sample collection and providing the information to the Ministry of Health that the Beta VOC is circulating in Equatorial Guinea based on the whole genome sequencing approach. By this time, the Beta VOC was already the dominant lineage. By using the L452R-specific RT-qPCR assay, the time from sample collection to providing the information of potential introduction of the Delta VOC was significantly reduced to <1 week. The first Delta VOC whole genome sequence was generated and uploaded to GISAID 8 weeks later.

We propose that in locations where SARS-CoV-2 sequencing capacity are limited and difficult to sustain, sequencing resources might be better utilized when focusing on phases in-between epidemiological waves which are characterized by lower infection rates. This might provide essential information on the introduction or emergence of new variants that might soon dominate the ensuing COVID-19 wave. Increase of sequencing efforts during times of high infection rates in the population might not yield novel sequencing information based on the strong dominance of distinct variants at that time.

We describe here the case of an asymptomatically infected adult male carrying two distinct lineages of SARS-CoV-2 that are both VOCs. The Beta VOC was first described in South Africa in October 2020 and has become the dominant lineage in many African countries by March 2021 (22). The Delta VOC was first described in India in October 2020 and has become the dominant lineage worldwide by August 2021 (23). Both VOC lineages have been circulating in Equatorial Guinea at the time when this co-infection was identified. Co-infection events between dominant SARS-CoV-2 lineages have been previously reported from the USA in 0.18% (53/29,993) of sequenced samples (24) and Brazil in 0.61% (9/1,462) of investigated samples (7). To our knowledge, this is the first time that a co-infection between the Beta and Delta VOCs is reported globally. Recombination among *Coronaviridae*, including SARS-CoV-2, has been described as an important evolutionary mechanism underlying genetic shift (9, 25). Newly recombined viruses might result in increased transmissibility or immune evasion and therefore continuous genomic monitoring of SARS-CoV-2 lineages are warranted (8, 22). Strengthening and continuous support of public health laboratories in Sub-Saharan countries to avoid underreporting of cases and enhance detection of emerging variants is a prerequisite for successful and global SARS-CoV-2 containment (20).

## Data Availability Statement

The datasets presented in this study can be found in online repositories. The names of the repository/repositories and accession number(s) can be found below: https://www.ncbi.nlm.nih.gov/, PRJNA770861; https://www.gisaid.org/, EPI_ISL_1672551–EPI_ISL_1672557, EPI_ISL_1673311–EPI_ISL_1673331, EPI_ISL_1700674–EPI_ISL_1700687, EPI_ISL_1753020–EPI_ISL_1753026, EPI_ISL_1989331, EPI_ISL_1989335, EPI_ISL_1989339, EPI_ISL_2002669–EPI_ISL_2002688, EPI_ISL_4601590–EPI_ISL_4601605, EPI_ISL_648303–EPI_ISL_648379, EPI_ISL_649155–EPI_ISL_649172, EPI_ISL_953402–EPI_ISL_953425.

## Ethics Statement

Sample collection and retrospective sequence analysis was conducted according to the guidelines of the Declaration of Helsinki and approved by the National Technical Committee for the Response and Monitoring of the Novel Coronavirus (Comité Tećnico Nacional de Respuesta y Vigilancia del Nuevo Coronavirus), which is charged with preventing, containing, controlling, tracking, and evaluating the development and evolution of COVID-19 in Equatorial Guinea. Informed consent for publishing the Beta and Delta VOC co-infection case was obtained from the patient. Publication of the SARS-CoV-2 epidemiological, genomic surveillance data and the co-infection case was additionally approved by the Equato-Guinean Ministry of Health and Social Welfare.

## Author Contributions

TS and CD: conceptualization, supervision, and project administration. SH, MM, PW, SR, and TS: methodology. SH, PW, and TS: software and validation. SH and TS: formal analysis, data curation, and visualization. SH, MM, EN, NB, TO, MO, PW, and UV: investigation. DM, MA, and WP: resources. SH, CD, and TS: writing—original draft preparation. All authors have read and agreed to the submitted version of the manuscript.

## Funding

The funding for this work was provided through the public–private partnership, the Equatorial Guinea Malaria Vaccine Initiative, supported by the Government of Equatorial Guinea, Ministries of Mines and Hydrocarbons, and Health and Social Welfare, Marathon Equatorial Guinea Production Limited, Noble Energy, Atlantic Methanol, Production Company, and the Equatorial Guinea Liquefied Natural Gas Company. The funder was not involved in the study design, collection, analysis, interpretation of data, the writing of this article or the decision to submit it for publication.

## Conflict of Interest

The authors declare that the research was conducted in the absence of any commercial or financial relationships that could be construed as a potential conflict of interest.

## Publisher's Note

All claims expressed in this article are solely those of the authors and do not necessarily represent those of their affiliated organizations, or those of the publisher, the editors and the reviewers. Any product that may be evaluated in this article, or claim that may be made by its manufacturer, is not guaranteed or endorsed by the publisher.
